# Dense Concentric Calcification in Kawasaki Disease With Early Graft Failure Successfully Treated With Rotatripsy-Assisted Angioplasty

**DOI:** 10.1016/j.jaccas.2026.107159

**Published:** 2026-03-06

**Authors:** Jegan Sivalingam, Jennifer Gough, Tushar Raina, Chrysovalantou Nikolaidou, Baskar Sekar

**Affiliations:** Department of Cardiology, Gloucestershire Hospitals NHS Foundation Trust, Gloucester, United Kingdom

**Keywords:** intravascular lithotripsy, Kawasaki disease, rotational atherectomy

## Abstract

**Background:**

The treatment of Kawasaki disease–related coronary artery disease (CAD) can be challenging due to long-standing medial calcification and aneurysmal remodeling.

**Case Summary:**

We present a case of a 32-year-old man with Kawasaki disease–related CAD, initially treated with coronary artery bypass graft surgery. The patient experienced early graft failure and presented with recurrent angina 13 months after his surgery. Repeat angiography revealed an occluded saphenous vein graft to the calcific right coronary artery. The right coronary artery was successfully treated with imaging-guided percutaneous coronary intervention using rotational atherectomy and intravascular lithotripsy.

**Discussion:**

This case underscores the value of tailored calcium modification and intracoronary imaging in managing Kawasaki disease–related CAD, especially in native vessels after graft failure.

**Take-Home Messages:**

Kawasaki disease–related CAD presents a clinical challenge in selecting the optimal revascularization strategy. Calcium modification and intracoronary imaging are crucial for a successful percutaneous coronary intervention in Kawasaki disease–related CAD.

## Visual Summary

**Figure:**
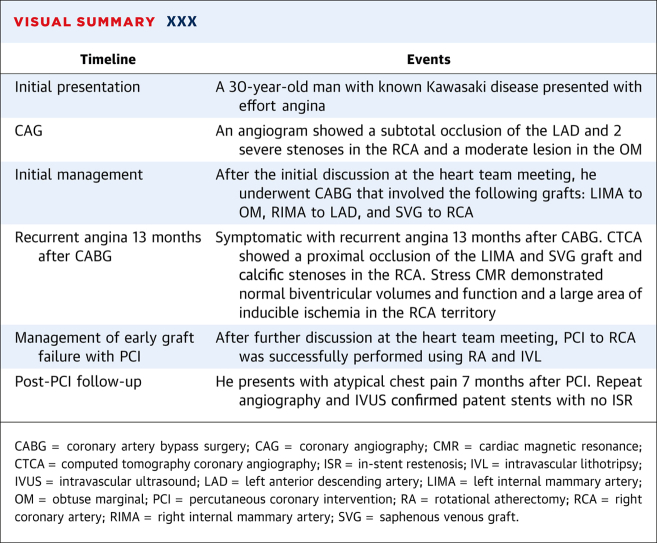

## Presentation

A 32-year-old man with known Kawasaki disease (KD) who had undergone coronary artery bypass graft surgery (CABG) 13 months earlier presented with recurrent episodes of effort angina with no ischemic changes on his electrocardiogram or elevation in his troponin T level.

**Take-Home Messages**

• This case illustrates the clinical dilemma in choosing the appropriate revascularization strategy in KD–related coronary artery disease.
• The use of appropriate calcium modification and intracoronary imaging during percutaneous coronary intervention for KD-related coronary artery disease is key to achieving a successful procedural outcome.

## Background

He was diagnosed with KD at the age of 11 years and was under regular rheumatology follow-up. He initially presented with effort angina at the age of 30 years. Coronary angiography was performed, which revealed a subtotal occlusion of his left anterior descending (LAD) artery, 2 severe stenoses in the right coronary artery (RCA), and a moderate stenosis in the obtuse marginal (OM). After discussion at the heart team meeting, he was referred for CABG. He received 3 bypass grafts including a right internal mammary artery (RIMA) to his LAD, a left internal mammary artery (LIMA) to his OM, and a saphenous venous graft (SVG) to his RCA.

## Differential Diagnoses

Troponin-negative chest pain occurring soon after CABG could be due to cardiac or noncardiac causes. The differential diagnoses considered were early graft failure, pericarditis, gastroesophageal reflux disease, and musculoskeletal chest pain.

## Investigations

**Figure 1:**
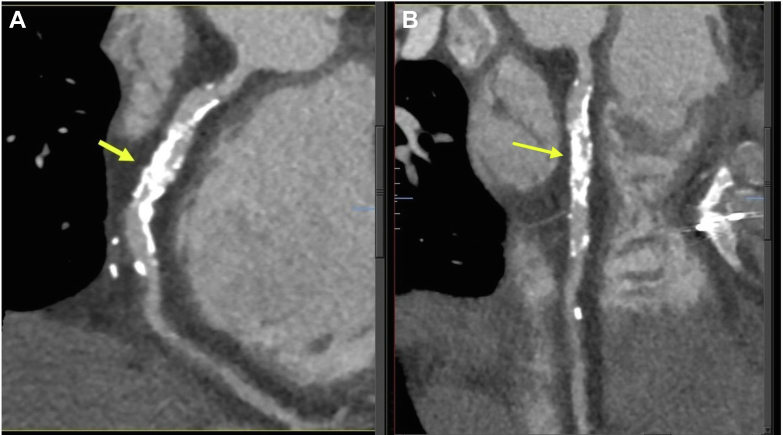
Computed Tomography Coronary Angiography Calcific stenoses of the right coronary artery: (A) left anterior oblique view and (B) longitudinal view.

Two-dimensional echocardiography revealed normal biventricular systolic function and no significant valvular pathology. Computed tomography coronary angiography showed a patent RIMA to the LAD, but a proximal occlusion of the LIMA to the OM and the SVG to the RCA. In addition, severe calcific stenosis was noted in the RCA (Figure 1), with only moderate calcific stenosis in the left circumflex.

Stress cardiac magnetic resonance demonstrated normal biventricular volumes and function with a large area of inducible ischemia in the RCA territory.

## Management and Procedural Details

**Figure 2:**
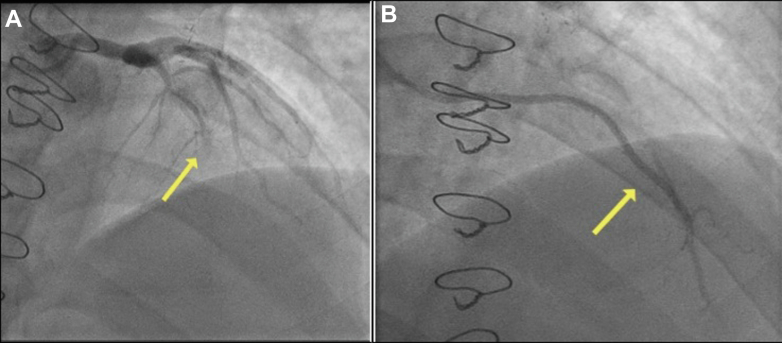
Post–Coronary Artery Bypass Graft Angiography (A) Occluded left anterior descending artery (LAD) and (B) patent right internal mammary artery to the LAD.

**Figure 3:**
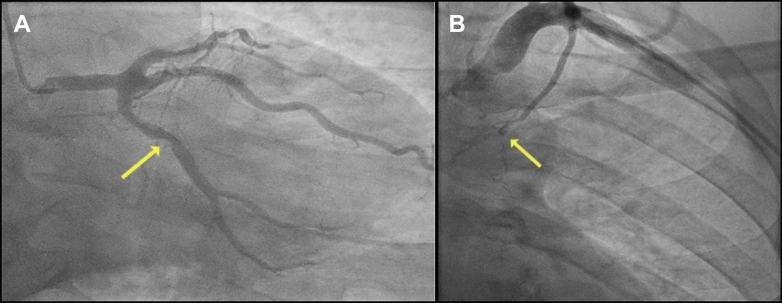
Post–Coronary Artery Bypass Graft Angiography (A) Moderate stenosis in the left circumflex and (B) occluded left internal mammary arteryto the obtuse marginal.

**Figure 4:**
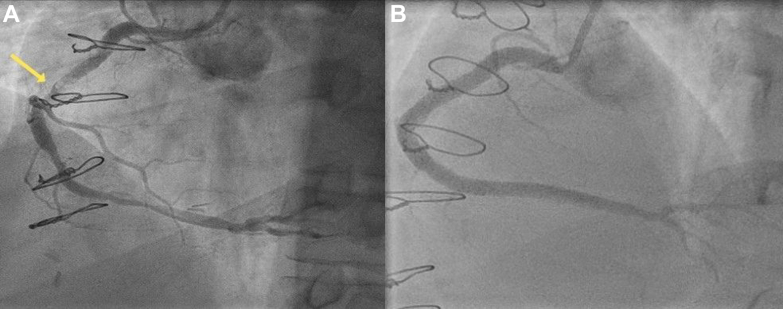
RCA Angiography (A) Pre-PCI showing severe calcific stenoses in the RCA and (B) post-PCI to the RCA. PCI = percutaneous coronary intervention; RCA = right coronary artery.

**Figure 5:**
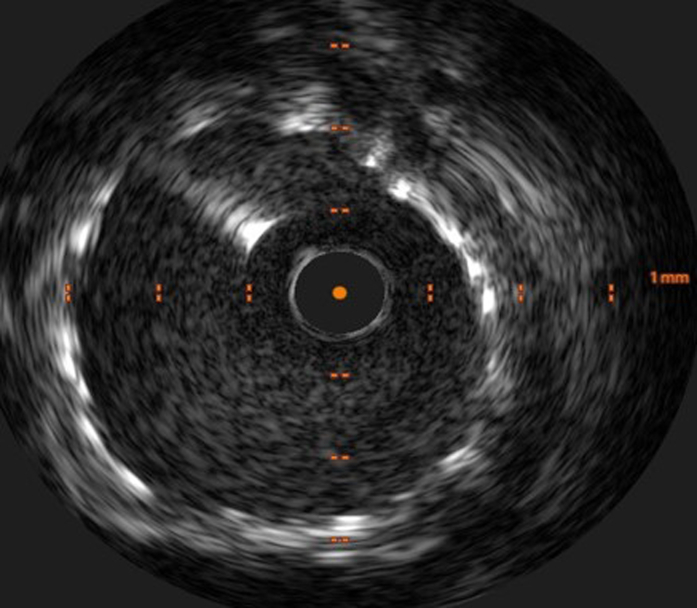
Intravascular Ultrasound After Percutaneous Coronary Intervention to the Right Coronary Artery

His case was discussed again at the heart team meeting, and the consensus was to offer him a percutaneous coronary intervention (PCI) to the RCA. During the procedural planning, it was decided that he would require adjunctive calcium modification given the circumferential long-segment calcification in his RCA noted on the 3-dimensional computed tomography coronary angiography reconstruction (Video 1). Transradial coronary angiography revealed an occluded LAD supplied distally by a patent RIMA (Figure 2), moderate disease in his left circumflex artery, and an occluded LIMA (Figure 3). The RCA had severe calcific stenoses in the proximal, mid, and distal segments (Figure 4). The RCA was engaged using a 6-F AL 0.75 guiding catheter (Medtronic Inc). A SION blue wire (Asahi Intecc) was placed in the distal RCA. Incremental predilatation was performed using a 1.0 × 5 mm Ryurei balloon (Terumo Corp), a 2.0 × 15 mm semicompliant balloon, and a 2.5 × 15 mm noncompliant balloon. Despite sequential balloon dilatation, the proximal stenosis did not yield. Rotational atherectomy (RA) (Boston Scientific) was therefore performed using a 1.5-mm burr at 160,000 rpm. Four short pecking passes were made, which encountered moderate resistance in the mid-RCA. Intravascular ultrasound (IVUS) was performed using the Eagle Eye Platinum IVUS catheter (Philips Volcano), which revealed residual concentric and eccentric calcification in the proximal and mid-RCA, respectively. Further calcium modification was undertaken using intravascular lithotripsy (IVL) using 80 pulses each of a 3.5 ×12 mm and a 4.0 ×12 mm Shockwave IVL balloon (Shockwave Medical Inc). After adequate lesion preparation, 5 everolimus-eluting Synergy Megatron (Boston Scientific Corp) drug-eluting stents measuring 4.0 × 32 mm, 4.0 × 24 mm, 4.0 × 20 mm, and 3.5 × 32 mm, 4.0 × 16 mm were implanted in an overlapping fashion. Postdilatation was performed using a 4.5 ×20 mm and a 5 ×15 mm noncompliant balloon. An excellent angiographic result was achieved (Figure 4), which was confirmed by a postprocedure IVUS examination (Figure 5), which revealed a minimal stent area of 19.9 mm^2^.

## Follow-Up

The patient was commenced on a 12-month course of ticagrelor at 90 mg twice daily in addition to long-term aspirin 75 mg once daily. He represented with atypical chest pain 7 months after the procedure. Repeat coronary angiography and an IVUS examination confirmed widely patent stents in the RCA with no in-stent restenosis.

## Discussion

KD is an idiopathic inflammatory vasculitis of medium-sized vessels, with a predilection for the coronary arteries.^1^ It is the most common cause of acquired coronary artery disease (CAD) in children with a worldwide incidence ranging from 5 to 22 per 100,000 of those aged 5 years or below.^2^ Of those who develop the illness, only 5% to 10% develop coronary aneurysms.^3^ Of these, 50% to 75% will develop aneurysms that do not regress.^3^ Long-term coronary sequelae from KD are rare with no randomized control trial data and only observational studies with limited sample size, available for decision-making.

## Pathogenesis

The pathology of KD-related coronary lesions is very different from the pathology of atherosclerotic CAD. KD-associated calcification is characteristically concentric, deep, and often extends longitudinally, reflecting medial myofibroblastic proliferation and osteogenic transformation rather than atherosclerotic necrotic core formation.^4^ This morphology results in markedly reduced vessel compliance, poor response to balloon angioplasty, and an increased risk of stent underexpansion if stenting is attempted without adequate lesion preparation and calcium modification.

## Treatment of Coronary Artery Disease in Kawasaki Disease

Current recommendations prefer CABG over PCI for KD-related CAD with Left Main involvement, multivessel CAD, chronic total occlusions, and those with reduced left ventricular ejection fraction or coexistent significant valvular pathology.^5^ Survival rates after CABG are generally good (9% mortality at 30 years), but recurrent cardiac event rates are common.^6^ The use of arterial grafts is recommended as SVG conduits have been reported to have significantly increased occlusion rates.^7^

In our case, the initial treatment with CABG was reasonable given the presence of multivessel CAD including a long segment of heavily calcified complex RCA disease. Early occlusion of both the SVG to RCA and LIMA to OM grafts is consistent with the reported poor graft patency in KD. A 20-year patency rate of 87% for internal thoracic artery grafts and 44% for vein grafts has been reported previously.^7^ Abnormal vessel remodeling, competitive flow dynamics, and preserved distal bed caliber are the potential contributing factors for graft failure.

PCI has been successfully used to treat KD-related CAD, with several case reports favoring the use of PCI in these patients. The complex anatomy and significant calcification often encountered in this patient cohort make PCI technically challenging.^4^ Stent malapposition, recurrent coronary aneurysms, and excessive in-stent restenosis have been reported after these procedures.^4^ The use of adjunctive tools such as IVUS, optical coherence tomography, RA, and IVL is therefore crucial to the successful outcome after PCI for KD-related CAD. RA followed by stenting had been shown to have a success rate of more than 90% in a Japanese case series.^4^ More recently, Hill et al^8^ have shown the safety and efficacy of IVL in severely calcified de novo coronary lesions with a success rate of >90%.

Our patient underwent a complex PCI to his RCA with an excellent and durable medium-term result. Adequate lesion preparation with multiple calcium-modifying techniques and the use of intracoronary imaging was key to the successful outcome of the procedure.

## Conclusions

Our case is unique in demonstrating early graft failure in the setting of KD-related CAD. The combined use of RA and IVL specifically tailored to KD-related calcium morphology is not widely reported. To the best of our knowledge, we could find only one other example in which this combination was used in this clinical context.^9^

## Funding Support and Author Disclosures

The authors have reported that they have no relationships relevant to the contents of this paper to disclose.

## Appendix

Video 1 3D Reconstruction of the CTCA showing extensive coronary calcification.
